# Successful simultaneous side by side placement of novel 6-Fr inside plastic stents for malignant hilar biliary obstruction

**DOI:** 10.1055/a-2808-7364

**Published:** 2026-03-09

**Authors:** Yoshinori Shimamoto, Hirotsugu Maruyama, Tatsuya Kurokawa, Yuki Ishikawa-Kakiya, Kojiro Tanoue, Yasuhiro Fujiwara

**Affiliations:** 1 12935 Department of Gastroenterology, Graduate School of Medicine, Osaka Metropolitan University, Osaka, Japan

Inside plastic stents (ISs) are widely used for malignant hilar biliary obstruction 1 2 . In Japan, 7-Fr ISs are typically used, and when multiple stents are required, they are inserted sequentially. However, the placement of the second and subsequent ISs can be challenging due to severe tumor-related strictures and interference with the previously placed stent. Problems associated with placing an additional IS include proximal migration 3 4 5 , the need for additional devices, and segmental cholangitis if the stent placement fails. Clinically, the occurrence of segmental cholangitis is a major issue as it requires repeated reinterventions and must therefore be avoided. Thus, endoscopists should aim for smooth stent deployment and timely completion of the procedure.

**Fig. 1:**
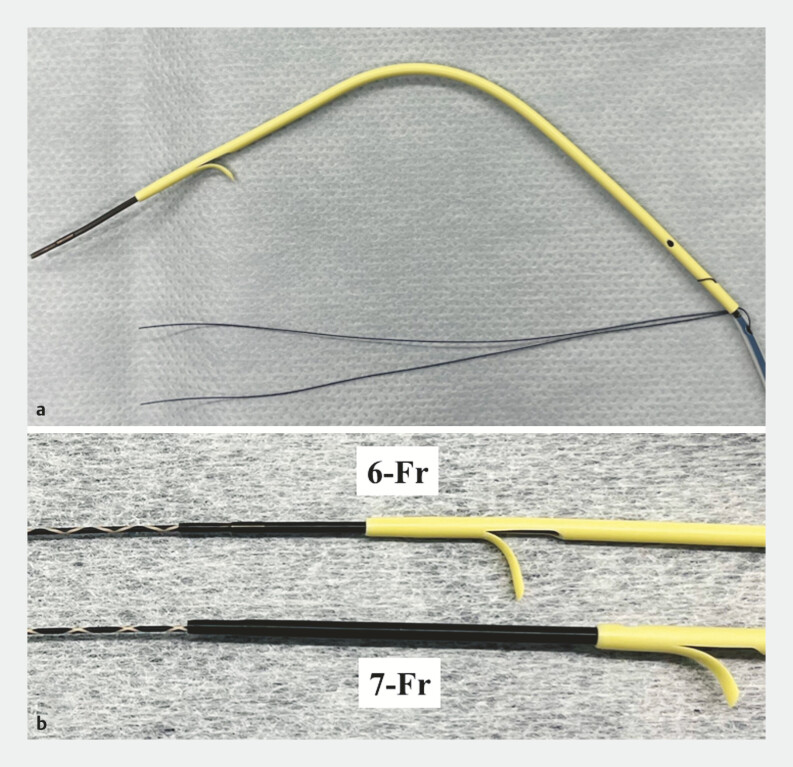
A new 6-Fr inside plastic stent. **a** This stent is an integrated delivery system with a thin, flexible, and middle-bend configuration. **b** The step differences at the interfaces between the guidewire (0.025-inch), inner catheter, and stent were minimal.

To meet these clinical needs, a new 6-Fr IS (Olympus Optical Co., Tokyo, Japan) was developed ( Fig. 1 ). Here, we report the successful simultaneous bilateral placement of this newly designed 6-Fr IS.

**Fig. 2:**
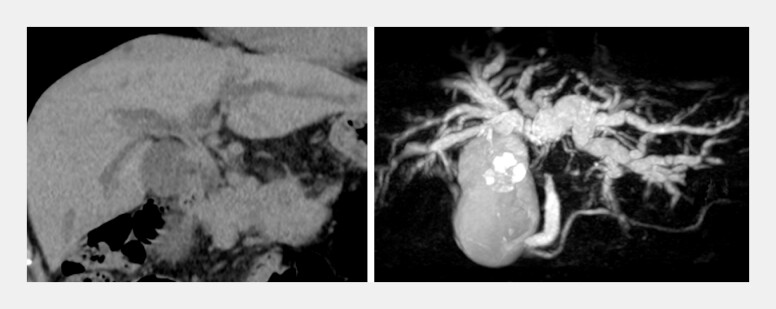
Computed tomography and magnetic resonance cholangiopancreatography revealed the wall thickening of the hilar bile duct accompanied by bilateral intrahepatic bile duct dilatation.

**Fig. 3:**
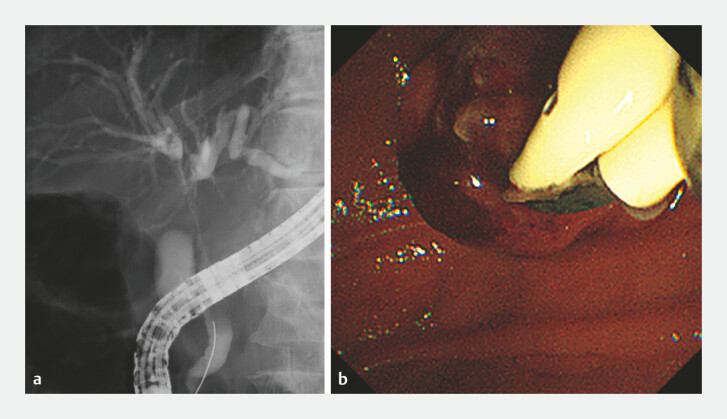
Stent placement. **a** Fluoroscopy demonstrated a bismuth type IIIa hilar obstruction. **b** Two novel 6-Fr inside plastic stents (ISs) were simultaneously introduced through the 4.2-mm working channel of a duodenoscope.

A 78-year-old man with malignant hilar obstruction secondary to cholangiocarcinoma was referred for jaundice. Computed tomography and magnetic resonance cholangiopancreatography revealed a hilar stricture with marked bilateral intrahepatic duct dilatation ( Fig. 2 ). During endoscopic retrograde cholangiopancreatography ( Video 1 ), a 0.025-inch guidewire was inserted into the common bile duct, and fluoroscopy demonstrated a bismuth type IIIa hilar obstruction. Guidewires were then advanced into the left and anterior bile ducts. Two novel 6-Fr ISs were simultaneously introduced through the 4.2-mm working channel of a duodenoscope and successfully deployed in parallel without interference between the stents or with the guidewires ( Fig. 3 ). The patient’s jaundice improved, and no adverse events occurred.

Simultaneous side-by-side deployment of novel 6-Fr inside plastic stents for malignant hilar biliary obstruction.Video 1

This IS has minimal steps between the stent, inner sheath, and guidewire, reducing insertion resistance and facilitating smooth passage through biliary strictures. Simultaneous parallel placement using this novel stent may shorten procedure time, improve technical success, and serve as a promising option for future practice.

Endoscopy_UCTN_Code_TTT_1AR_2AZ Endoscopy_UCTN_Code_CCL_1AZ_2AC
